# HIV and early hospital readmission: evaluation of a tertiary medical facility in Lilongwe, Malawi

**DOI:** 10.1186/s12913-018-3050-9

**Published:** 2018-04-02

**Authors:** Kashmira Satish Chawla, Nora E. Rosenberg, Christopher Stanley, Mitch Matoga, Alice Maluwa, Cecilia Kanyama, Jonathan Ngoma, Mina C. Hosseinipour

**Affiliations:** 1 The University of North Carolina Project-Malawi, Tidziwe Centre, Private Bag A-104, Lilongwe, Malawi; 2 The Department of Medicine, Division of Infectious Diseases, University of North Carolina, 130 Mason Farm Rd. (Bioinformatics), CB# 7030, Chapel Hill, NC 27599-7030 USA; 3 The Department of Medicine, Kamuzu Central Hospital, P.O. Box 149, 265 Lilongwe, Malawi

**Keywords:** Readmissions, Sub-Saharan Africa, Quality, HIV, Malawi

## Abstract

**Background:**

Delivery of quality healthcare in resource-limited settings is an important, understudied public health priority. Thirty-day (early) hospital readmission is often avoidable and an important indicator of healthcare quality.

**Methods:**

We investigated the prevalence of all-cause early readmission and its associated factors using age and sex adjusted risk ratios (RR) and 95% confidence intervals (CI). A retrospective review of the medical ward database at Kamuzu Central Hospital in Lilongwe, Malawi was conducted between February and December 2013.

**Results:**

There were 3547 patients with an index admission of which 2776 (74.4%) survived and were eligible for readmission. Among these patients: 49.7% were male, mean age was 39.7 years, 36.1% were HIV-positive, 34.6% were HIV-negative, and 29.3% were HIV-unknown. The prevalence of early hospital readmission was 5.5%. Diagnoses associated with 30-day readmission were HIV-positive status (RR = 2.41; 95% CI: 1.64–3.53) and malaria (RR = 0.45; 95% CI: 0.22–0.91). Other factors associated with readmission were multiple diagnoses (excluding HIV) (RR = 1.52; 95% CI: 1.11–2.06), and prolonged length of stay (≥ 16 days) at the index hospitalization (RR = 3.63; 95% CI: 1.72–7.67).

**Conclusion:**

Targeting HIV-infected inpatients with multiple diagnoses and longer index hospitalizations may prevent early readmission and improve quality of care.

## Background

Delivery of quality healthcare in resource limited settings is an important public health priority. Quality healthcare, defined as safe, effective, patient-centered, timely, efficient, and equitable [1] care is associated with improved patient outcomes [2, 3]. Early hospital readmission (returning within 30 days of discharge from an index hospitalization) is an indicator of healthcare quality [4]. Early readmissions are often avoidable and commonly reflect inadequate or incomplete treatment during the first hospitalization or poor coordination of services at the time of discharge [4–7]. Early readmissions, which increase healthcare costs and burden individuals, have been well characterized in developed countries [8].

In sub-Saharan Africa, early readmission is not well characterized. A range of quality-related concerns have been described, such as misdiagnosis, poor communication during handover, non-adherence to evidence-based diagnostic and treatment guidelines, and safety violations [9–11]. However, few assessments of readmission [12–20] have been conducted, and research on such healthcare indicators is needed to guide quality improvement [21].

Additionally, the disease burden in Sub-Saharan Africa differs from developed countries. Importantly, HIV is highly prevalent in the region and many HIV-infected patients experience high morbidity and mortality, even after the scale-up of combination antiretroviral therapy (cART) [22, 23]. In previous assessments of readmission in the region, HIV-infected patients were more likely to be readmitted. However, these assessments did not focus on *early* readmission [24], were conducted before the introduction of cART rollout [25], or among specific subpopulations [19], before significant ART roll-out. Understanding predictors of early readmission in a large tertiary referral environment after cART rollout is an important area to explore.

At Kamuzu Central Hospital (KCH), a tertiary hospital in Lilongwe, Malawi, we analyzed readmission patterns in the inpatient medicine wards. We described the prevalence of all- cause early readmission, assessed factors associated with early readmission, characterized readmission diagnoses, and explored the impact of HIV/AIDS on all readmission.

## Methods

### Sample and setting

KCH is a 1000 bed facility that serves a catchment area of 5 million people. Each year 46,000 adults are first admitted to the medical short stay, the triage department for non-trauma related acute cases. From here, most are managed and discharged, and approximately 5000 are admitted to the general medicine wards [26]. The short stay and medical wards are staffed by 9 nurses and 4 medical teams. Each team consists of one consultant, two registrars, one clinical officer, and 2 interns [27].

Admission to the hospital and all medical services are provided at no cost, including medication. An onsite laboratory has the capability for blood cultures, chemistry, transfusion services, microscopic analysis, and testing for common infectious diseases. Radiology services such as x-ray, ultrasound, and CT scans are also available. These diagnostic modalities are on-site, though often unavailable, due to lack of supplies or staff. Lastly, an on campus housing facility is also available for family members or other caretakers, which reduces pressure for discharge from patients or family members.

Routine HIV testing was conducted in the wards by trained HIV testing and counseling (HTC) counselors using opt-out procedures described previously [23]. Briefly, the counselors systematically screened all patients admitted to the wards. Patients with a previous unknown HIV status or a negative HIV test (> 3 months ago) were offered opt-out HTC by the HTC counselor (serial testing with Determine HIV 1/2 and Unigold HIV 1/2) [23, 27]. The results of the test were then recorded in the patient’s file and health passport book.

### KCH medical wards database

Patient information was routinely collected in paper patient files. Information from these files was then abstracted by study ward clerks using a paper data abstraction tool. The abstraction tool includes name, age, hospital ID number, gender, date of admission, hospitalization outcome, date of outcome, and select clinical information. Clinical information included HIV status of index admission, HTC result, up to four admission diagnoses (not including HIV), and up to four discharge diagnoses. Data abstractors classified admission and discharge diagnoses into one of sixty common conditions based on clinician notes. One of these diagnoses was sepsis. Due to lack of routine blood culture tests, the diagnosis of sepsis was largely made on clinical grounds. HIV positive patients with a history of fever often received a diagnosis of sepsis if the rapid malaria test was negative, there were no chest findings, and there was improvement with empiric broad spectrum antibiotic treatment. This is the standard of practice in under-resourced healthcare facilities in Malawi. These abstracted data were then entered into the KCH Medical Wards Access database by a research assistant [23].

### Identification of readmissions

A retrospective review of this database was conducted in February 2014 to determine the prevalence and predictors of readmission. All patients 14 years and older, with an initial admission between February 1, 2013 and November 30, 2013 were included in the analysis. This presumed first hospitalization in the medical ward was defined as an index admission. A readmission was defined as a subsequent hospitalization in the medical ward between February 2 and December 31. Follow-up time was administratively censored on December 31, 2013. It was possible that patients who appeared to be presenting for the first time in early 2013 were actually patients presenting for readmission from a 2012 index admission. In order to assess potential misclassification of the index admission, we conducted a sensitivity analysis excluding all index admissions from February.

Linkage of the index admission and readmission records was conducted manually by study staff and investigators. Two or more database records with identical hospital IDs, names, and ages were considered linked admissions. Records with similar information on these three variables (*N* = 101/4057, 2.5%) were adjudicated by clinical staff, who often remembered the patients, and a trained research assistant. Typically, if discrepancies seemed like clerical errors, such as reversal of digits or misspellings, the original data abstraction tool or patient file was consulted. Time to readmission was calculated as the number of days between the date of the outcome from the index hospitalization and the date of entry at readmission or censorship at the end of the study period. Readmission of < 30 days were defined as “early readmission,” the primary outcome.

### Variables

HIV status was the primary factor of interest. Persons were classified as HIV-infected if an HIV-positive status was known at the time of admission (known positive) or if a new HIV-positive result was documented during the index hospitalization (new positive). HIV-uninfected was defined as known HIV-negative status within three months at the time of admission or a new HIV-negative result documented during the index hospitalization. Patients were classified with an unknown HIV status if the HIV status was unknown at the time of admission and no HIV status was documented during the index hospitalization.

**Fig. 1:**
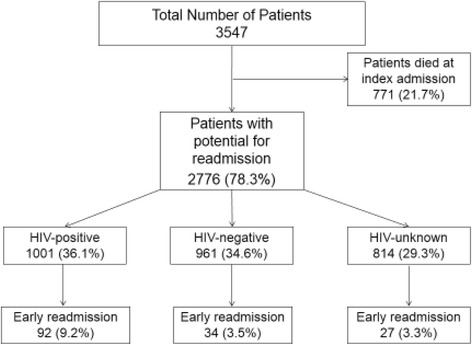
Flow diagram of the study. The total number of patients admitted and eligible for readmission in the adult medicine wards over a period of 10 months. There were 153 early (30-day) hospital readmissions, stratified by HIV status

Other variables included in the analysis included age, gender, index hospitalization outcome, index discharge diagnoses, co-morbidity, and length of stay at the index hospitalization. Index and readmission hospitalization outcomes were categorized as discharge, abscond, transfer, or death. Persons who died during the index hospitalization were not included in the readmission analysis (Fig. 1). Multiple diagnoses were classified based on the discharge diagnosis list. This list did not include HIV status. Persons with two or more diagnoses were classified as having multiple diagnoses. Length of stay (LOS) was defined as the time period between the date of admission of the index hospitalization and the date of death, discharge, abscond, or transfer. This variable was categorized as 0–1 day, 2–7 days, 8–15 days, and ≥ 16 days. The ten most prevalent discharge diagnoses were each categorized into dichotomous variables.

### Data analyses

Among those at risk for early readmission, we compared the distribution of age, gender, and length of stay by HIV status using chi-squared tests. Risk factors of early readmission were assessed using generalized linear models with a log link and binomial distribution to estimate risk ratios. We implemented unadjusted models and a multivariate adjusted model. To arrive at the adjusted model, we first included all variables that had *p*-values < 0.2 in unadjusted analysis. We then retained those variables that had *p*-values < 0.05 in the full multivariate model. Using these same variables, we also conducted an analysis stratified by HIV status (HIV-infected versus HIV-uninfected patients) to assess whether these factors of interest differed by HIV status. Finally, we assessed whether HIV status was associated with mortality at the readmission hospitalization.

We also explored time to readmission beyond thirty days. Kaplan-Meier curves were generated to depict time to readmission by HIV status (negative, positive, and unknown) and compared using log rank tests. We implemented Cox proportional hazards regression models to assess whether factors of interest were associated with time to readmission.

## Results

### Characteristics

A total of 3547 patients had at least one hospitalization during the 10 months of observation (Fig. 1). Most (*N* = 2646, 74.8%) were discharged at their index hospitalization, 63 (1.78%) absconded, 60 (1.69%) were transferred, 7 (0.20%) had a missing outcome, and 771 died (21.7%) and were therefore not at risk for readmission. A total of 2776 patients were at risk for readmission (Table 1). Of these patients, the mean age was 39.7 years (SD 16.5), 49.7% were males, and the median LOS was 4 days (IQR, 2–7). Distribution of HIV status was 36.1% HIV-positive, 34.6% HIV-negative, and 29.3% HIV-unknown. The distribution of age, outcome, and length-of-stay differed by HIV status (Table 1).

**Table 1:** Characteristics of Patients at Risk of Readmission by HIV Status (*N* = 2776)

Characteristics	HIV-positive (1001)	HIV-negative (*n* = 961)	HIV-unknown (*n* = 814)	*P*-value
	N, %	N, %	N, %	
Gender				
Male	475 (47.5)	481 (50.1)	425 (52.2)	
Female	526 (52.6)	480 (50.0)	389 (47.8)	0.127
Age (years)				
14–20 yrs	33 (3.3)	124 (12.9)	91 (11.2)	
21–30 yrs	204 (20.4)	231 (24.0)	171 (21.0)	
31–40 yrs	377 (37.7)	184 (19.1)	161 (19.8)	
41–50 yrs	191 (19.1)	105 (10.9)	96 (11.8)	
50+ yrs	126 (12.6)	237 (24.7)	233 (28.6)	
Missing^b^	70 (7.0)	80 (8.3)	62 (7.6)	< 0.0001
Outcome (index admission)^a^				
Discharge	947 (94.6)	922 (95.9)	777 (95.5)	
Abscond	17 (1.7)	16 (1.7)	30 (3.7)	
Transfer	36 (3.6)	17 (1.8)	7 (0.9)	
Missing	1 (0.1)	6 (0.6)	0	< 0.0001
LOS (index admission)^a^				
0–1 days	99 (9.9)	127 (13.2)	181 (22.2)	
2–7 days	626 (62.5)	611 (63.6)	512 (62.9)	
8–15 days	214 (21.4)	165 (17.2)	86 (10.6)	
≥ 16 days	61 (6.1)	58 (6.0)	34 (4.2)	
Missing	1 (0.1)	0	1 (0.1)	< 0.0001

^a^*LOS* length of stay in the hospital during a hospitalization, ^b^Missing values are included when calculating percentages.

Demographic, clinical outcomes and hospitalization characteristics are described and compared by HIV status

### Early readmission

One hundred fifty three patients (5.5%) experienced an early readmission. Forty six patients had a second readmission, thirteen had a third readmission, five had a fourth readmission, and one had a fifth readmission. Of patients with a second readmission, 18 had a second readmission within 30 days; 6 had a third readmission within 30 days; 1 had a fourth readmission within 30 days. The median time to early readmission was 11 days (IQR, 7–19 days). At the first early readmission, 63.4% were discharged, 34.0% died, and 0.7% absconded at their readmission hospitalization.

Among early readmissions, 84 (55%) were known HIV-infected persons, 8 (5%) were newly diagnosed HIV-infected persons, 34 (22%) were HIV-uninfected, and 27 (18%) had an unknown HIV status. Persons who were HIV-infected were more likely to experience an early readmission (9.2%) than those who were HIV-uninfected (3.5%) or with an unknown HIV status (3.3%) (*p* < 0.0001).

**Fig. 2:**
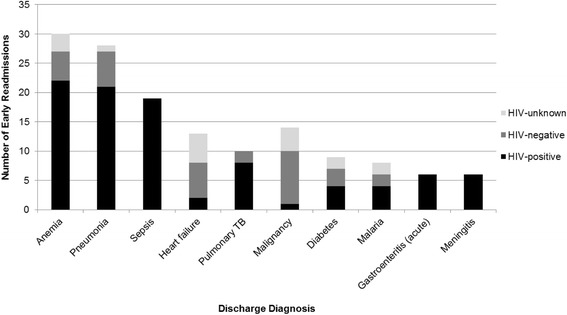
Early Readmission, by HIV Status, for the 10 Most Common Discharge Diagnoses (*N* = 153). Diagnoses illustrate that a large proportion of the early readmissions were HIV–positive in many of the discharge diagnosis categories. *TB = tuberculosis

The ten most prevalent discharge conditions were anemia, malaria, sepsis, pneumonia, heart failure, pulmonary tuberculosis, diabetes, malignancy, acute gastroenteritis, and meningitis (Fig. 2). The readmission rates ranged from 1.8% for malaria to 10% for malignancy. HIV prevalence also varied substantially among readmitted patients with a range of 7.1% among those with malignancy to 100% for sepsis, acute gastroenteritis, and meningitis.

### Risk of early readmission

Results of unadjusted and adjusted analyses are reported in Table 2. In the adjusted analysis, age and gender were not associated with risk of early readmission (Table 2). Malaria (RR = 0.45, 95% CI: 0.22, 0.91) was the only clinical diagnosis associated with decreased risk of early readmission. Patients with multiple diagnoses had an increased risk of early readmission (RR = 1.52, 95% CI: 1.11, 2.06) compared to those with only one condition. The longer the LOS at the index admission, the higher the risk of early readmission (RR = 3.63, 95% CI: 1.72, 7.67) comparing ≥ 16 days to ≤ 1 day). When we excluded February readmissions, no coefficient changed by more than 10%, suggesting our results were robust to the index classification.

**Table 2:** Factors Associated with Early (30-day) Readmissions (*N* = 2776)

Factors	Unadjusted RR (95% CI)*	*P*-value	Adjusted RR (95% CI)**	*P*-value
Age				
14–20 yrs	1			
21–30 yrs	1.32 (0.71, 2.42)	0.37		
31–40 yrs	1.03 (0.56, 1.90)	0.92		
41–50 yrs	1.36 (0.72, 2.58)	0.34		
50–99 yrs	0.99 (0.53, 1.86)	0.98		
Gender				
Male	1			
Female	1.00 (0.74, 1.36)	0.99		
HIV status				
Negative	1		1	
Positive	2.60 (1.77, 3.81)	< 0.0001	2.41 (1.64, 3.53)	< 0.0001
Unknown	0.94 (0.57, 1.54)	0.80	1.06 (0.64, 1.73)	0.83
Discharge Diagnoses				
Anemia	1.77 (1.21, 2.60)	< 0.0001		
Pneumonia	1.24 (0.83, 1.84)	0.30		
Sepsis	0.90 (0.56, 1.44)	0.66		
Heart failure	1.49 (0.87, 2.59)	0.15		
Malaria	0.35 (0.17, 0.71)	0.01	0.45 (0.22, 0.91)	< 0.0001
Pulmonary TB	1.38 (0.74, 2.56)	0.31		
Malignancy	3.30 (2.00, 5.48)	< 0.0001		
Diabetes	1.41 (0.74, 2.69)	0.30		
Gastroenteritis	0.54 (0.24, 1.21)	0.14		
Meningitis	0.63 (0.28, 1.39)	0.25		
Multiple discharge diagnosis (non-HIV)				
No	1		1	
Yes	1.53 (1.12, 2.08)	0.01	1.52 (1.11, 2.06)	0.01
Length of stay				
0–1 days	1		1	
2–7 days	1.96 (1.02, 3.73)	0.04	1.56 (0.82, 3.00)	0.17
8–15 days	3.50 (1.77, 6.91)	< 0.0001	2.43 (1.22, 4.84)	0.01
≥ 16 days	5.05 (2.41, 10.62)	< 0.0001	3.63 (1.72, 7.67)	< 0.0001
Outcome (Index Admission)				
Discharge	1			
Abscond	1.15 (0.44, 3.01)	0.78		
Transfer	0.91 (0.30, 2.76)	0.86		

*RR Risk Ratio, **Adjusted Risk Ratio based on a multivariable model that variables are still significant at *p* < 0.05.

Table 2 shows the multivariable relationship between HIV-positive status and early readmission

In analyses stratified by HIV status, we found very similar results to our main analyses (Table 3). Multiple discharge diagnoses remained associated with higher early readmission in HIV-infected patients, although estimates were less precise.

**Table 3:** Factors Associated with Early (30-day) Readmissions (*N* = 2776), Stratified by HIV Status

	HIV Negative		HIV Positive	
Factors	Adjusted RR (95% CI)	*P*-value	Adjusted RR (95% CI)	*P*-value
Discharge Diagnoses				
Malaria	0.55 (0.13, 2.39)	0.43	0.43 (0.16, 1.17)	0.10
Multiple discharge diagnosis (non-HIV)				
Yes	1.21 (0.54, 2.75)	0.64	1.51 (1.01, 2.24)	0.04
Length of stay				
2–7 days	1.41 (0.40, 4.94)	0.54	1.47 (0.60, 3.58)	0.40
8–15 days	3.58 (0.92, 13.9)	0.07	1.75 (0.68, 4.50)	0.25
> 16 days	4.13 (0.83, 20.59)	0.08	2.65 (0.95, 7.42)	0.06

*RR* Risk Ratio, *CI* Confidence interval

Table 3 shows the relationship between multiple discharge diagnoses, length of stay, malignancy, malaria, anemia and early readmission, stratified by HIV status

In both unadjusted and adjusted analyses, being HIV-positive was associated with an increased risk of early readmission (RR = 2.60 and RR = 2.41). Patients with an HIV-unknown status had a similar risk of early readmission as HIV-negative patients when adjusted (Table 2). A separate adjusted analysis showed that HIV-positive patients who knew their HIV status at index admission were 2.17 times more likely to have an early readmission compared to those who learned their HIV-positive status at the index hospitalization (95% CI: 1.08, 4.42).

**Fig. 3:**
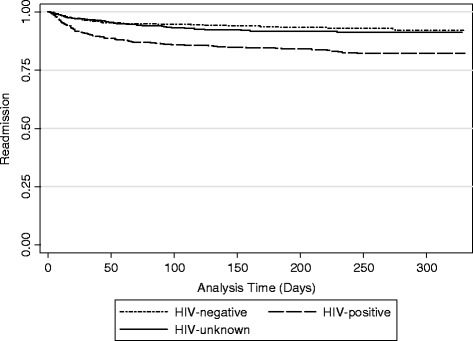
Time to Readmission by HIV Status. Kaplan–Meier curves show the time to readmission following all-cause index hospitalization, over a period of 10 months. There was a difference in the time to readmission by HIV status (*p* < 0.0001)

Patients who were HIV-positive were also more likely to have earlier time to readmission than those who were HIV-negative or HIV-unknown status (*p* < 0.0001) (Fig. 3). The results of unadjusted Cox proportional hazard regression were similar to the unadjusted risk model: HIV positive status (HR = 2.63, 95% CI: 1.96, 3.54), anemia (HR = 1.68, 95% CI: 1.24, 2.27), malignancy (HR = 2.77, 95% CI: 1.74, 4.42), multiple diagnoses (HR = 1.26, 95% CI: 1.00, 1.60), and LOS > 16 days (HR = 2.88, 95% CI: 1.69, 4.91).

Patients who were HIV-positive at early readmission were 1.80 times as likely to die at the readmission visit as those who were HIV-negative (95% CI: 0.95, 3.39). Those with HIV-unknown status were somewhat less likely to die than those who were HIV-negative (RR = 0.62, 95% CI: 0.32, 1.20), but these results were not statistically significant.

## Discussion

Our study is among the first to investigate all-cause early readmissions among medicine inpatients in a resource-limited tertiary healthcare facility in Sub-Saharan Africa, in the era of cART rollout. We found that a substantial proportion of patients eligible for readmission were HIV-positive (36.1%) and that these persons were more than twice as likely to experience an early readmission. In addition, HIV-positive patients were readmitted earlier, and were more likely to die at the readmission hospitalization. Multiple diagnoses, and prolonged length of stay during the index hospitalization were also associated with an increased risk of early readmission. The only clinical diagnosis associated with decreased risk of early readmission was malaria.

Our finding that HIV infection is associated with early readmission is consistent with other assessments. HIV infection was found to be associated with readmission among all-cause medicine [24] and pediatric malaria patients in other low-resource settings [19]. Elevated readmission rates were also documented among HIV-infected inpatients in this setting, but prior to ART scale-up [25].

There are a few possible explanations for our findings of elevated readmission for HIV-infected patients. First, HIV is a chronic disease that requires involved management. Patients who are on HAART may experience side effects and toxicity, treatment failures, and immune reconstitution syndrome [24] which may result in readmission. Next, low adherence to treatment predisposes the patient to AIDS-related morbidities [24, 28–32]. Sub-optimal management of such conditions during the initial hospitalization may force HIV-positive patients to return to the hospital shortly after discharge. Another possibility is poor transition of care from the inpatient setting to self-management at home due to weak linkage to outpatient care [33]. Lastly, cessation of therapy post-discharge, due to lack of follow-up, may have led to further deterioration of their medical condition and re-hospitalization. Additional information on disease status and patient management may elucidate the most plausible explanation for this finding. It is necessary to further investigate and identify specific shortcomings in in-hospital practices which contribute to inadequate HIV inpatient care [34].

Multiple diagnoses were also associated with increased risk of early readmission. Multiple diagnoses have been implicated as a predictor for 30-day readmission among general medical patients [35, 36], patients with acute myocardial infarction [37], community acquired pneumonia [38], or heart failure at index hospitalization [39]. Patients with multiple diagnoses are usually more complex. Inadequate management of multiple conditions during hospitalization may lead to complications post-discharge [6, 36]. In addition, the acute illness superimposed on an underlying condition may cause a relapse in the immediate post-hospitalization time period [36]. Lastly, persons in under-resourced settings may experience greater difficulties with management of multiple conditions in an unsupported home environment post-discharge [36]. These challenges with care inside the hospital and transition to self-care at home may amplify chances of readmission.

Prolonged LOS was also associated with increased risk of early readmission. This assumed a dose-response relationship: the longer someone stayed in the hospital, the more likely they were to return. This is comparable to some studies, which have demonstrated a positive relationship [40], and contrasts with other studies which demonstrate an inverse relationship [28, 41, 42]. In our setting, longer LOS in the hospital may be a proxy for delays in the execution of care due to low staff to patient ratios, bed availability, disjointed flow, lack of access to supplies, and other organizational factors that inhibit optimal care [10]. Alternatively, this association could reflect severity of disease or time needed to manage multiple disease conditions, particularly among HIV-infected persons [28].

Readmission rates in developed nations range from 1.1% - 25% [8]. Studies among subpopulations of patients in Africa reported readmission rates between 8.5% - 70% [12–20], higher than our 30-day readmission rate of 5.5%. However these studies contain longer readmission intervals up to 1 year, which may help account for the difference.

Similarly, the proportion of HIV-infected patients that were readmitted early (9.2%) in our study was lower than US estimates of over 19% [28–30]. A number of reasons may explain the lower than expected readmission rate. Patients in our setting face high barriers to accessing care, such as transport and lack of supportive care outside the hospital, which hinders their return to the hospital [43]. Some patients may have died before they could present for readmission, which may actually reflect a high post-discharge mortality [44]. Next, some patients may have sought care at other facilities, rather than returning to a tertiary facility. Lastly, some may not have been captured due to difficulty with record linkage.

Early readmission is a single indicator that does not provide a complete understanding of quality of care. In fact, the discordance between early readmission and other quality markers, such as 30-day mortality, further underscores the limitations of this indicator [45]. Future studies should assess multiple quality indicators, such as markers of process of care, in conjunction with early readmission [2, 45, 46]. This will more fully inform quality of care and also augment our understanding of specific clinical activities that could be targeted for improvement.

Record linkage was challenging in this setting due to an inconsistent unique identifier. We matched patient records based on hospital ID number, name, and age. Ward clerks and the data personnel were consulted on any possible matches, which were resolved by looking up the file, but some patients may have been erroneously classified. We may have under-estimated or over-estimated early readmission due to missed matches or false matches of readmissions.

There were several analyses that we were not able to conduct. We were not able to conduct a review of patient files to distinguish avoidable versus unavoidable readmissions. Information on factors such as severity of disease during the index hospitalization, measure of comorbidity, cART use and initiation, outpatient follow-up, and patterns of referrals for readmission are needed to understand their impact on readmission.

Markers of quality of inpatient care and hospital discharge practices are lacking in low and middle income countries, particularly for HIV-infected patients [34]. Our study sought to address this knowledge gap with the characterization of early readmissions in a medical facility in a low-resource, high HIV prevalence setting. While the overall frequency of early readmission was low, it provides a reference value for this indicator for similar settings in the region. We learned that interventions to reduce readmission should target inpatients with multiple disease conditions, prolonged LOS during index hospitalization, and HIV infection. The assessment of early readmission provides critical information that can guide strategies to optimize medical care activities and transition of care, particularly for HIV-positive inpatients [34].

## Conclusion

The study shows high early hospital readmission amongst HIV positive patients with multiple diagnoses, and prolonged length of stay during the index hospitalization. The study further shows the need to optimize inpatient medical care and linkages to outpatient care to avoid readmission and associated mortality amongst HIV positive patients. Therefore, strategic interventions targeting HIV-infected inpatients with multiple diagnoses and longer index hospitalizations are needed to prevent early readmission and improve quality of care.

## Abbreviations

cART: antiretroviral therapy
CI: confidence intervals
HIV: Human Immunodeficiency virus
HTC: HIV testing and counseling
KCH: Kamuzu Central Hospital
LOS: Length of stay
RR: adjusted risk ratios
